# Tryptophan pathway profiling in multiple sclerosis patients treated with ocrelizumab

**DOI:** 10.3389/fimmu.2025.1603663

**Published:** 2025-06-09

**Authors:** Carolina Ricci, Matteo Pivetta, Eleonora Martinis, Viviana Valeri, Carolina Colliva, Nicola Giacchè, Simone Lorenzut, Daniela Cargnelutti, Barbara Frossi, Silvia Tonon

**Affiliations:** ^1^ Department of Medicine, University of Udine, Udine, Italy; ^2^ Tes Pharma S.r.l., Corciano, Italy; ^3^ Neurology Unit, “Head, Neck and Neurosciences” Department, University Hospital of Udine, Udine, Italy

**Keywords:** multiple sclerosis, ocrelizumab, tryptophan, kynurenine, serotonin, indole

## Abstract

**Introduction:**

L-Tryptophan (Trp) metabolism is impaired across various chronic inflammatory pathologies, including Multiple Sclerosis (MS). Trp processing relies on three metabolic routes, namely Kynurenine, Serotonin and Indole pathways. The host microbiota significantly impacts Trp metabolism, primarily by being responsible for Indole metabolites production and secondarily by shaping both Kynurenine and Serotonin pathways. Pathological conditions and pharmaceutical treatments can elicit changes in microbial populations, leading to alterations in metabolites production and therefore determining rearrangements in host metabolism. Currently, no simultaneous exploration and comparison of all three Trp related metabolic routes has been performed in the context of MS patients before and after Ocrelizumab (OCR) treatment.

**Methods:**

By performing mass spectrometry on plasma samples collected from healthy controls and MS patients before and six months after OCR treatment we provided a comparative investigation of Trp metabolomics profile.

**Results and discussion:**

Our data points out to concurrent alterations of Trp-related pathways among both OCR treated and untreated MS patients. Furthermore, MS treated patients presented a pattern resembling health state for various metabolites across the pathways. The results reported in our research may contribute to unveiling new perspectives and understanding regarding MS pathogenetic mechanisms.

## 1 Introduction

Multiple sclerosis (MS) is a chronic inflammatory autoimmune disease that affects the central nervous system (CNS). It is characterized by the targeting of myelin, which results in axonal and neuronal damage, and consequently leads to a wide range of neurological symptoms. This pathology is known to affect the female sex more frequently than male (with a ratio 4:1 approximately) (1). The precise etiology of MS is not clear, but it is well established that environmental factors contribute to its pathogenesis, including diet and gut commensal microbiota composition (2–5). This can be attributed to the well-established role of nutrients and microbiota-derived metabolites in shaping host’s immunity (6). Notably, dietary interventions have been shown to exert immunomodulatory effects, influencing the balance between pro- and anti-inflammatory responses. For example, in preclinical model of MS, namely experimental autoimmune encephalomyelitis (EAE), intermitted fasting was demonstrated to alter gut microbiota, suppress the proinflammatory T helper 17 (Th_17_) response and induce anti-inflammatory regulatory T cells (T_regs_) (7). In line with this evidence, a 3-month long fasting mimicking diet led to improve quality of life in Relapsing Remitting MS (RRMS) patients (7).

Conversely, it is known that the pathogenetic process of MS and the therapeutic strategies employed may influence the metabolism of various factors introduced through the diet (8): patients under pharmacological treatment display increased microbiota diversity and decreased presence of pro-inflammatory bacteria (8), probably playing a role in shaping the course of disease (9).

For all the reasons outlined above, it is crucial to study in more detail the metabolism of dietary nutrients in relation to gut microbiota composition and the use of drugs for MS, to leverage diet- and microbiota-based interventions for therapeutic benefits.

This work focuses on L-Tryptophan (Trp), one of the nine essential amino acids, which can only be introduced by diet. At the intestinal lumen level, Trp is metabolized by the gut microbiota through the Indole (Ind) pathway, while the absorbed Trp is utilized in the Kynurenine (Kyn) and Serotonin (also referred to as 5-hydroxytryptamine (5-HT)) pathways. Absorbed Trp is employed in protein synthesis or processed either by host-enzymes through Kyn and 5-HT pathways. Approximately 90-95% of absorbed Trp is metabolized through Kyn pathway by Tryptophan-2,3-dioxygenase (TDO) in the liver and Indoleamine-2,3-dioxygenase (IDO) that is expressed by several cells, including immune cells (10). The remaining is converted to 5-HT mainly by enterochromaffin cells of intestinal mucosa by Tryptophan hydroxylase 1 (TPH1) (11). Differently, Ind pathway starts in the lumen of large intestine, where specific commensal bacterial strains release Trp from ingested proteins and further process it into Ind-derivatives which can be transferred to blood (12). Specifically, bacterial species, namely *Lactobacillus, Bacteroidetes, Anaerostipes, Bifidobacterium*, and *Clostridium*, take part in this metabolic route. Trp conversion to Tryptamine (Tryp) is operated by *Clostridium* sp*orogenes* and *Ruminococci* (13, 14), while *Lactobacilli* converts Trp into Indole-3-Carboxaldehyde (I3A) (15). Furthermore, *Clostridium* sp*orogenes* catalyzes the Trp conversion into Indole-3-Propionic Acid (IPA) (16). Microbiota can also influence 5-HT pathway acting on enterochromaffin cells (17) and Kyn pathway by modulating the activity of IDO (18). Ultimately, microbiota could influence the Trp availability for absorption (19).

It has been demonstrated that MS patients have an altered microbiota composition compared to healthy individuals and that some treatments can alter proportions between different bacteria strains (5). Specifically, the treatment with ocrelizumab (OCR), a monoclonal antibody directed against CD20 in use since 2018 (20), has been correlated to changes in MS-microbiota (21).

Our study focused on analyzing the levels of circulating Trp and its metabolites in both healthy individuals and RRMS patients before and after treatment with OCR. The final goal was to assess how MS pathogenesis affects Trp metabolism and to determine whether OCR therapy can help reverse any alterations observed in MS patients.

## 2 Materials and methods

### 2.1 Sample collection patients’ characteristics

Blood samples were collected in vial with Sodium Citrate and centrifugated for 10 min at 800g to isolate plasma, then stored at -80°C. The study was approved by the regional ethical committee of Friuli Venezia Giulia (Italy), CEUR-2021-Os-139. In this study we enrolled a total of 10 healthy controls (HC), 4 female and 6 male (mean age 31.7 ± 5.5), and 17 patients diagnosed with RRMS, 12 females and 5 males (mean age 39 ± 8.4). Patients underwent through a washout from previous therapy and received OCR after this period and had a mean Expanded Disability Status Scale (EDSS) of 2 ± 1.5.

### 2.2 Measurement of Trp metabolites

The quantification of Trp metabolites was performed using liquid chromatography-tandem mass spectrometry (UPLC-ESI-MS2) in positive electrospray ionization (ESI+ MS) mode (22). The separation was achieved using an Acquity HSS T3 column (2.1 × 100 mm, 1.8 µm, Waters) with a gradient elution over 11 minutes. Mobile phases consisted of 0.2% formic acid in water and methanol, with a flow rate of 400 μL/min and an injection volume of 5 μL.

The ESI source conditions included a desolvation temperature of 500°C, a desolvation gas flow of 1000 L/hr, a source temperature of 150°C, and a capillary voltage of +2.7 kV. Quantification was based on a standard calibration curve (0.0025–50 μM) using multiple reaction monitoring (dMRM) mode.

### 2.3 Analytical equipment and reagents

High-purity solvents (99.98%) were used, including UPLC-grade water (Milli-Q), methanol (Chebios), and formic acid (Sigma-Aldrich). The instrument setup consisted of an ACQUITY UPLC H-Class Bio module coupled to a Waters Xevo TQD triple quadrupole MS. Analyte standards were sourced from Sigma-Aldrich. The following compounds were analyzed: L-Trp (L-Tryptophan); L-Kyn (L-Kynurenine); KYNA (Kynurenic Acid); AA (Anthranilic Acid); 3-OH-Kyn (3-HydroxyKynurenine); XA(Xanthurenic Acid); 3-OH-AA (3-HydroxyAnthranilic Acid); QA (Quinolinic Acid); NAM (Nicotinamide); MNAM (1-Methylnicotinamide chloride); 5-HT (Serotonin (5-HydroxyTryptamine)); 5-MT (Melatonin); 5-HTP (5-Hydroxy-L-Trp); 5-HIAA (5-Hydroxy-Indole-Acetic Acid); I3AA (3-Indoleacetic acid); I3A (Indole-3-Carboxaldehyde); IPA (Indole-3-Propionic Acid). The internal standard QA-3D (Quinolinic acid 4,5,6-D3) was included in the analysis to ensure accuracy and reproducibility of quantification.

### 2.4 Sample preparation

Plasma samples were processed by adding ice-cold acidified methanol containing the internal standard (Quinolinic acid (4,5,6-D3)) for protein precipitation. Samples were sonicated for 30 minutes, centrifuged at maximum speed for 10 minutes, and the supernatant was evaporated to dryness. The residue was reconstituted in 50 μL of 0.2% formic acid in water before LC-MS/MS analysis.

### 2.5 Stock and working solutions

Stock solutions (100 mM) of Trp metabolites were prepared in methanol and stored at -80°C. Working solutions were prepared by serial dilution to obtain calibration (0.0025–50 μM) and quality control (0.025, 0.5, 25 μM) samples, stored in glass vials at -80°C.

### 2.6 Calibration curves

Seven-point calibration curves were constructed. The calibration curve range was 0.0025–50 μM. Linear regression analysis (1/x2 weighting) was used to determine analyte concentrations. Quality control samples were prepared at three levels: low (0.25 μM), medium (0.5 μM), and high (2.5 μM).

### 2.7 UPLC-ESI-MS/MS analysis

Chromatographic separation was performed using a UPLC HSS T3 column with a gradient elution profile (0–90% B over 5 minutes, followed by re-equilibration). The column temperature was maintained at 30°C. The mass spectrometer operated in Multiple Reaction Monitoring (MRM) mode with optimized parameters for each metabolite, ensuring sensitivity and selective quantification. Retention times and mass transitions were recorded for all Trp pathway metabolites.

### 2.8 Heatmap generation for Trp-related pathways

Heatmaps were generated for visualizing the distribution of Trp-related metabolites in Kyn, 5-HT and Ind pathways among HC, MS patients before OCR treatment (MS-preOCR) and MS patients after six months of OCR treatment (MS-postOCR) samples groups. Raw metabolites quantification data were divided into three different matrixes, each for the three Trp-related pathways, with rows representing the individuals and columns representing each metabolite. Matrixes were firstly normalized through z-score normalization and then grouped by means for each sample group. Heatmaps were generated using R Software (v4.4.2; R Core Team 2024) through the “gplots” package (v3.2.0; Warnes G et al.), using “heatmap.2” function and by scaling per columns. A “PiYG” color palette from “RColorBrewer” package (v1.1-3; Neuwirth E.) was used to represent the data values for each metabolite: color ranging from pink to green was used to represent the z-scores, with color pink indicating the lower z-score and green the higher. Dendrograms were added to illustrate the clustering patterns.

### 2.9 Statistical analysis

All datasets were tested for normality before applying parametric or non-parametric test (Mann-Whitney test was used for non-parametric analysis). For HC *vs* MS-preOCR and HC *vs* MS-postOCR unpaired tests were used, instead when analyzing MS-preOCR *vs* MS-postOCR a paired test was applied. All the statistical analysis were performed using GraphPad Prism 10.4.1.

## 3 Results

### 3.1 AA, QA and XA are impaired in Kyn pathway among MS patients

**Figure 1:**
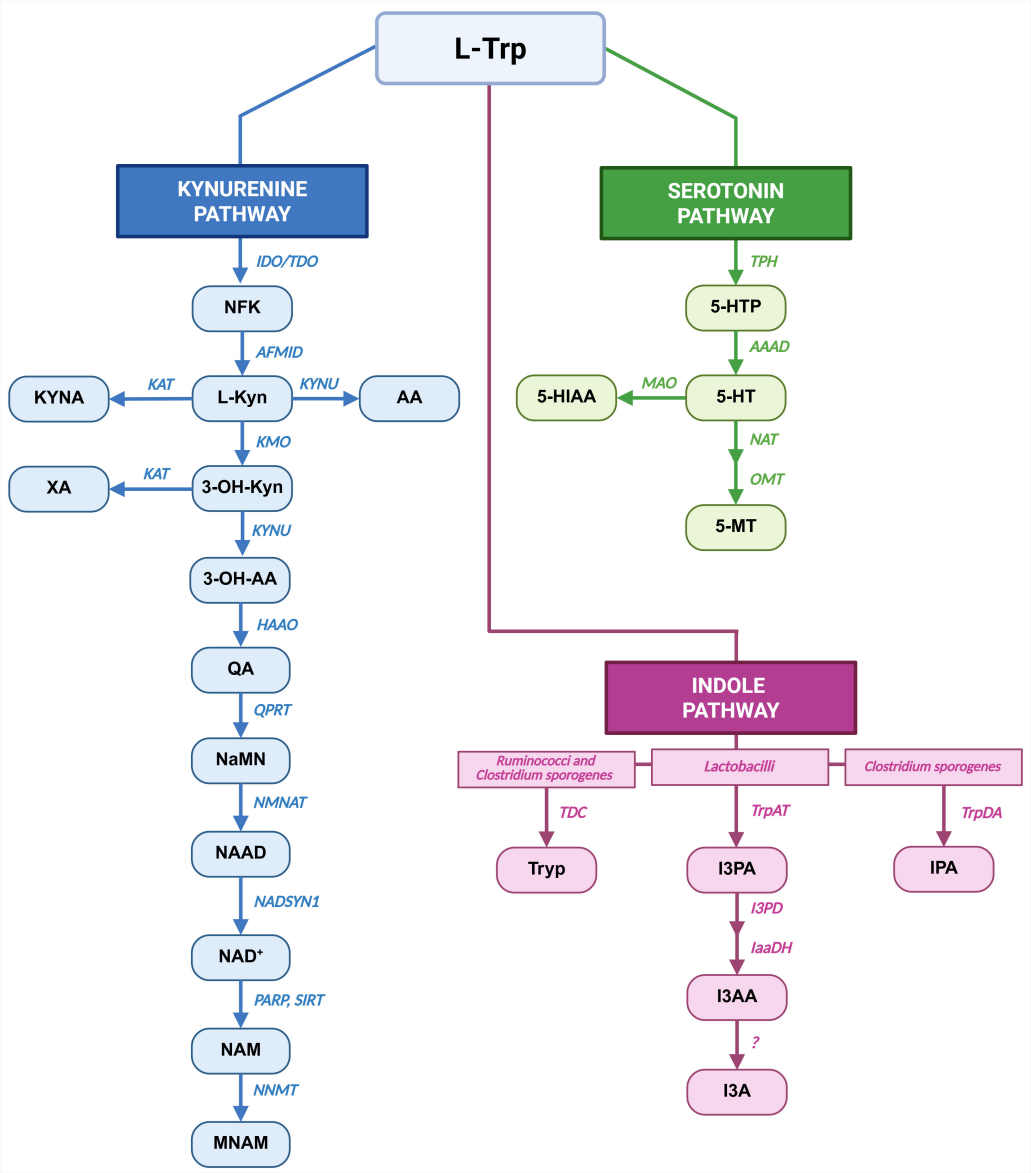
Pathways of Trp metabolism. Main metabolic pathways responsible for Trp metabolism: Kyn pathway in blue, 5-HT pathway in green and Ind pathway in pink are shown. For each metabolic route the metabolites are indicated as blocks, while the enzymes and bacterial strains are in italics. Created in BioRender. Created in BioRender. Pivetta, M. (2025) https://BioRender.com/yafet2l.

**Figure 2:**
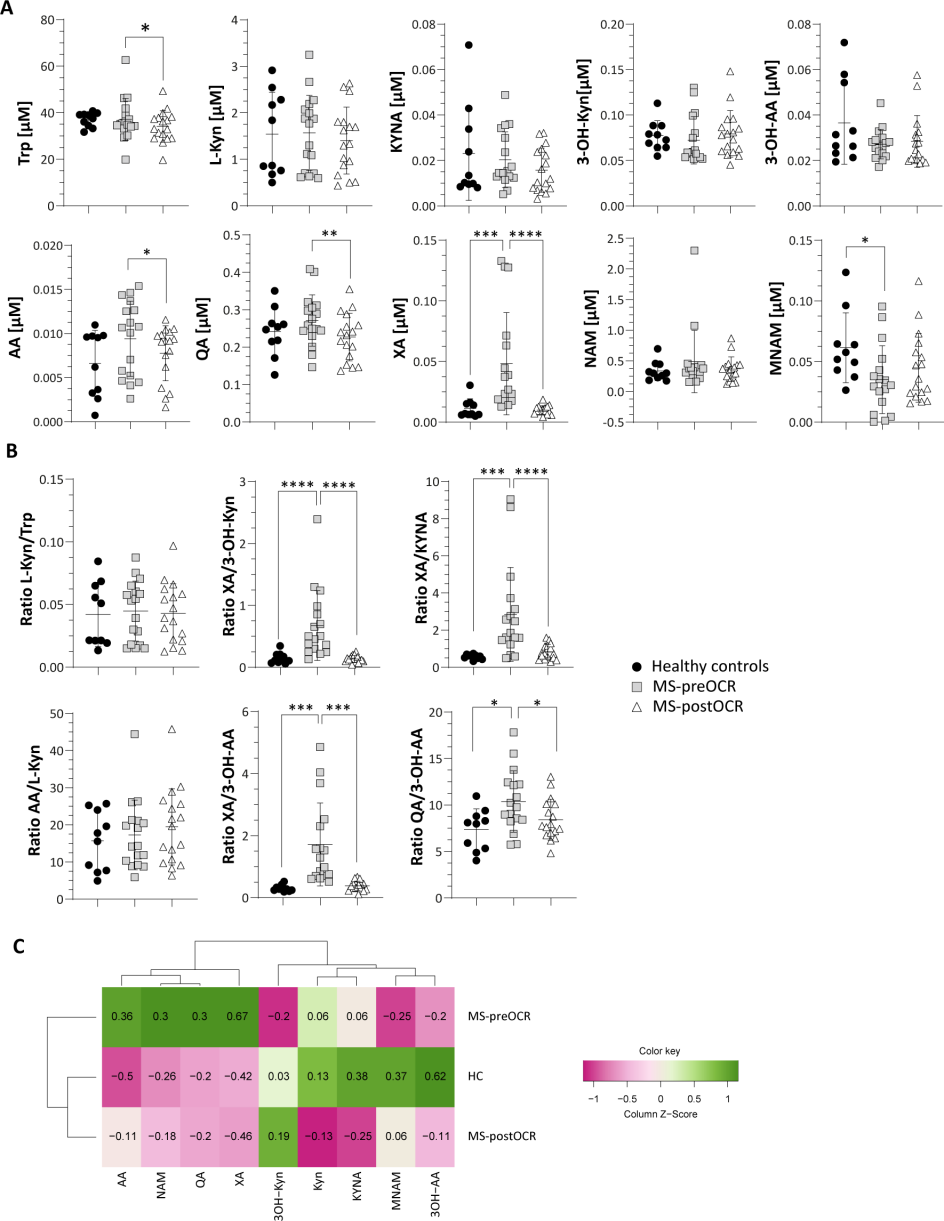
Evaluation of Kynurenine Pathway in HC and MS patients before and after OCR treatment. **(A)** Micromolar [µM] concentration of circulating Trp and Kyn metabolites (L-Kyn, KYNA, 3-OH-Kyn, 3-OH-AA, AA, QA, XA, NAM and MNAM) **(B)** L-Kyn/Trp ratio for the evaluation of IDO/TDO activity; XA/3-OH-Kyn ratio for the evaluation of KAT2; XA/KYNA ratio for the evaluation of KAT activity; AA/L-Kyn ratio; KYNA/L-Kyn ratio for the evaluation of KAT activity; 3OK-Kyn/L-Kyn ratio for the evaluation of KMO activity; XA/3-OH-AA ratio to evaluate the production of XA over 3-OH-AA from 3-OH-Kyn. XA/KYNA ratio for the evaluation of KAT activity; XA/3OH-Kyn ratio for the evaluation of KAT2; QA/3-OH-AA ratio to evaluate the production of QA over 3-OH-AA. **(C)** Heatmap including all Kyn-related metabolites among HC and MS before and after OCR treatment. Heatmap’s rows indicate the condition, while the columns indicate the metabolites of Kyn-related pathway. Dendrograms indicating clustering groups are reported among rows and columns. The color-legend for z-score values is reported. MS patients (n= 17) before (MS-preOCR) and after (MS-postOCR) OCR treatment; Healthy controls (HC; n= 10). HC *vs* MS-preOCR or MS-postOCR: non-parametric Mann-Whitney unpaired test; MS-preOCR *vs* MS-postOCR: non-parametric paired test. *p<0.05, **p<0.01, ***p<0.001, ****p < 0.0001.

Trp metabolites (shown in **Figure 1** and listed in **Table 1**) were assayed in all the three conditions: healthy controls (HC), MS patients before OCR treatment (MS-preOCR) and MS patients after six months of OCR treatment (MS-postOCR). To determine whether there was an imbalance in Trp metabolism between HC, MS-preOCR, and MS-postOCR conditions, we first evaluated the total plasma Trp concentration among groups. We found a significant decrease in Trp levels in MS-postOCR group and MS-preOCR (**Figure 2A**). We next evaluated the Kyn pathway considering that the concentration of a metabolite can be either associated to an accumulation from its non-utilization or an augmented activity of the enzymes associated with its production. We observed that L-Kyn levels are comparable in the three groups (**Figure 2A**). Similarly, the L-Kyn/Trp ratio, which is indicative of IDO/TDO enzymatic activity, is comparable among the conditions (**Figure 2B**). The concentrations of Kynurenic Acid (KYNA), 3-Hydroxykynurenine (3-OH-Kyn) and 3-Hydroxyanthranilic acid (3-OH-AA) were similar among HC, MS-preOCR and MS-postOCR groups (**Figure 2A**). Conversely, Anthranilic Acid (AA) and Quinolinic Acid (QA) concentrations were higher in MS-preOCR group compared with MS-postOCR group and the same trend can be appreciated between HC and MS-postOCR group (**Figure 2A**). We found instead that Xanthurenic Acid (XA) concentration was significantly higher in the MS-preOCR group when compared with both HC and MS-postOCR individuals (**Figure 2A**).

**Table 1:** Trp metabolites and related enzymes.

Metabolite	Enzyme	Trp-related pathway
Trp: L-Tryptophan		
NFK: N’-formylkynurenine	*IDO*: Indoleamine-2,3-dioxygenase	Kyn pathway
L-Kyn: L-Kynurenine	*TDO*: Tryptophan-2,3-dioxygenase	
KYNA: Kynurenic Acid	*AFMID*: Arylformamidase	
AA: Anthranilic acid	*KAT*: Kynurenine aminotransferase	
3-OH-Kyn: 3-Hydroxykynurenine	*KYNU*: Kynureninase	
XA: Xanthurenic acid	*KMO*: Kynurenine 3-monooxygenase	
3-OH-AA: 3-Hydroxyanthranilic acid	*HAAO*: 3-hydroxyanthranilate 3,4-dioxygenase	
QA: Quinolinic acid	*QPRT*: Quinolinate phosphoribosyl transferase	
NaMN: Nicotinic Acid Mononucleotide	*NAPRT*: Nicotinate phosphoribosyltransferase	
NA: Nicotinic Acid	*NMNAT*: Nicotinamide-nucleotide adenylyltransferase	
NAAD: Nicotinic Acid Adenine Dinucleotide	*NADSYN1*: NAD synthetase	
NAD^+^: Nicotinamide Adenine Dinuclotide	*PARP*: poly(ADP-ribose) polymerase	
NAM: Nicotinamide	*SIRT*: Sirtuin	
NMN: β-Nicotinamide mononucleotide	*NAMPT*: Nicotinamide phosphoribosyltransferase	
MNAM: 1-Methylnicotinamide	*NNMT*: Nicotinamide N-methyltransferase	
5-HTP: 5-Hydroxy-L-tryptophan	*TPH*: Tryptophan hydroxylase	5-HT Pathway
5-HT: 5-Hydroxytryptamine (also known as Serotonin)	*AAAD*: Aromatic L-amino acid decarboxylase	
5-HIIA: 5-Hydroxy-Indole-Acetic Acid	*MAO*: Monoamine oxidase	
5-MT: Melatonin	*NAT*: N-Acetyltransferase	
	*OMT*: O-Methyltransferase	
Tryp: Tryptamine	*TDC*: Tryptophan decarboxylase	Ind Pathway
I3PA: Indole-3-pyruvic Acid	*TrpAT*: Tryptophan aminotransferase	
IPA: Indole-3-propionic Acid	*I3PD:* Indolepyruvate decarboxylase	
I3A: Indole-3-carboxaldehyde	*IaaDH*: Indoleacetaldehyde dehydrogenase	
I3AA: 3-Indoleacetic acid	*TrpDA*: Tryptophan decarboxylase	

The XA/3-OH-Kyn ratio (**Figure 2B**) reflects Kynurenine Aminotransferase 2 (KAT2) isoform activity, that mediates the XA synthesis from 3-OH-Kyn (23). This ratio is higher in MS-preOCR patients compared with the other groups, suggesting that KAT2 is more active.

The XA/KYNA ratio, which indicates a preference for XA or KYNA production from the common precursor L-Kyn, is higher in MS-preOCR, suggesting a shift in metabolic production towards XA rather than KYNA (**Figure 2B**). On the contrary, AA/L-Kyn ratio is equal across conditions (**Figure 2B**), implying a possible accumulation of AA. Moreover, by evaluating XA/3-OH-AA and QA/3-OH-AA ratios (**Figure 2B**), we can speculate that there is a disbalance of 3-OH-AA production in favor of XA and QA in MS-preOCR patients with respect to both HC and MS-postOCR. Finally, the Kyn pathway culminates in Nicotinamide Adenine Dinucleotide (NAD^+^) synthesis (**Figure 1**), that is further metabolized in Nicotinamide (NAM) and then as 1-Methylnicotinamide (MNAM), which we found to be present with lower concentrations in MS-preOCR patients (**Figure 2A**) when compared to HC. The overall alterations in Kyn pathway emerged in the heatmap (**Figure 2C**) and, as can be observed, HC and MS-postOCR cluster together.

### 3.2 5-HT pathway is dysregulated in RRMS patients

**Figure 3:**
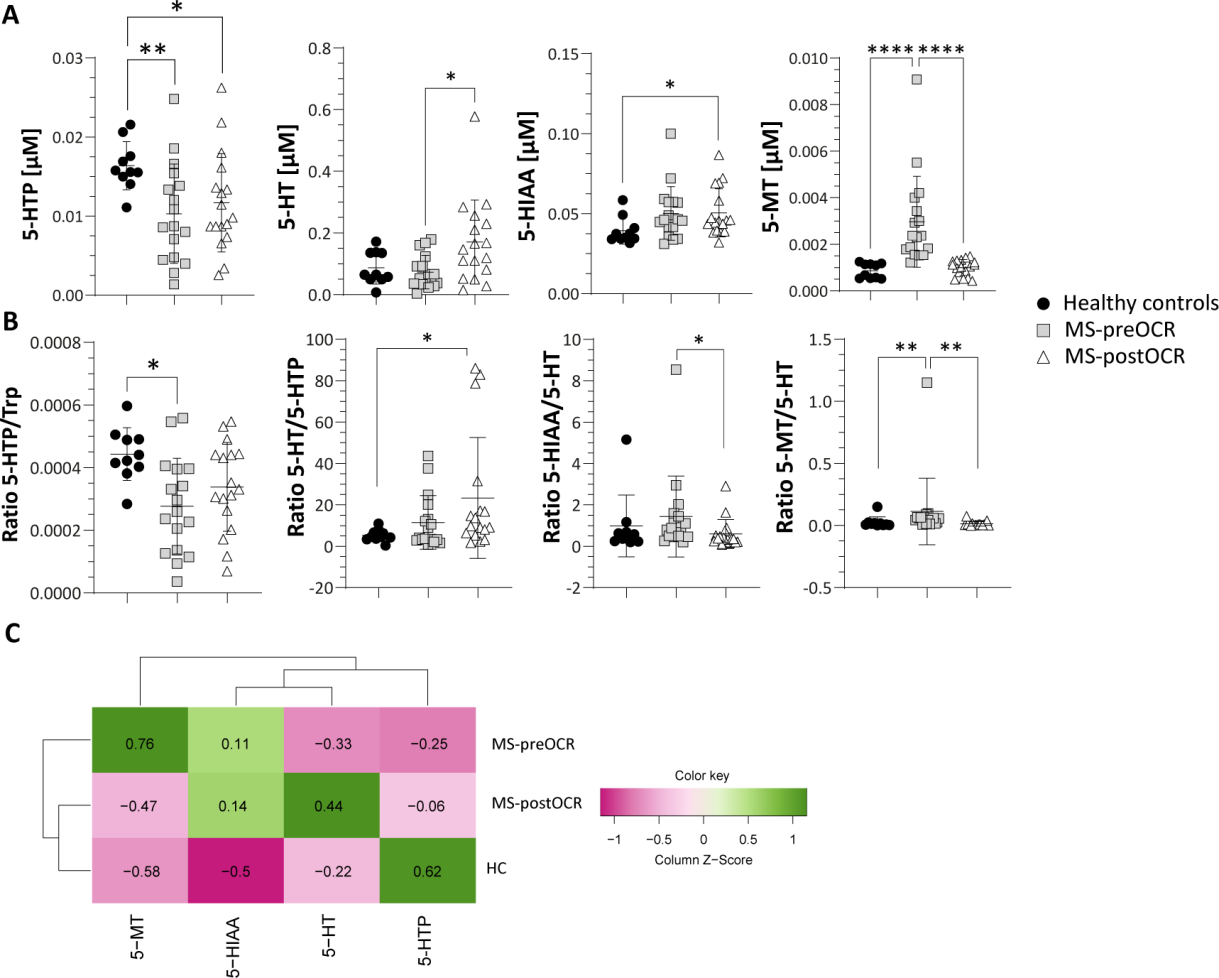
Evaluation of Serotonin pathway in HC and MS patients before and after OCR treatment. **(A)** Micromolar [µM] concentration of 5-HT metabolites (5-HTP, 5-HT, 5-HIAA and 5-MT). **(B)** 5-HTP/Trp ratio for the evaluation of TPH1 activity; 5-HT/5HTP ratio for the evaluation of AAAD; 5-HIAA/5-HT ratio for the evaluation of MAO; 5-MT/5-HT ratio for the evaluation of NAT and OMT. **(C)** Heatmap including all 5-HT-related metabolites among HC and MS before and after OCR treatment. Heatmap’s rows indicate the condition, while the columns indicate the metabolites of 5-HT-related pathway. Dendrograms indicating clustering groups are reported among rows and columns. The color-legend for z-score values is reported. MS patients (n= 17) before (MS-preOCR) and after (MS-postOCR) OCR treatment; Healthy controls (HC; n= 10). HC *vs* MS-preOCR or MS-postOCR: non-parametric Mann-Whitney unpaired test; MS-preOCR *vs* MS-postOCR: non-parametric paired test. *p<0.05, **p<0.01, ***p<0.001.

The absorbed Trp can also be directed into the 5-HT metabolic route. The 5-HT pathway relies on the action of TPH1 enzyme, which converts Trp into 5-Hydroxy-L-tryptophan (5-HTP), the metabolic precursor of 5-HT. As shown in **Figure 3A**, 5-HTP concentration is significantly lower in both MS-preOCR and postOCR patients than in HC. The significant and slight decrease in 5-HTP/Trp ratio in MS-preOCR and the tendency towards diminished levels postOCR respectively suggests a possible defect in TPH1 activity.

Moreover, we observed a significant increase in 5-HT in MS-postOCR patients compared to both MS-preOCR and HC, respectively (**Figure 3A**), along with a similar trend for the 5-HT/5-HTP ratio (**Figure 3B**), which reflects . the These data suggest thatAromatic L-amino acid decarboxylase (AAAD) enzymatice activity. These data suggest that AAAD appears to be is more active after treatment. 5-Hydroxy-Indole-Acetic Acid (5-HIAA) is higher in MS-postOCR (**Figure 3A**), although 5-HIAA/5-HT ratio is similar to HC and decreased compared with MS-preOCR group (**Figure 3B**). These findings can be attributed to the increase in 5-HT after OCR, rather than an alteration in Monoamine oxidase (MAO) enzymatice activity, expressed by 5-HIAA/5-HT ratio. The 5-HT pathway converges on Melatonin (5-MT) metabolite: we found its concentration to be higher in MS-preOCR patients over both HC and MS-postOCR (**Figure 3A**), and this phenomenon could be dependent on both an increased activity of N-Acetyltransferase (NAT) and O-Methyltransferase (OMT) enzymes, since also 5-MT/5-HT ratio is increased in this same group (**Figure 3B**). Collectively, these data indicate a global alteration of the 5-HT pathway in MS patients and, as observed before for the Kyn pathway, HC and MS-postOCR cluster together in the heatmap (**Figure 3C**).

### 3.3 Microbiota-dependent Ind pathway is impaired in I3A production in MS patients

**Figure 4:**
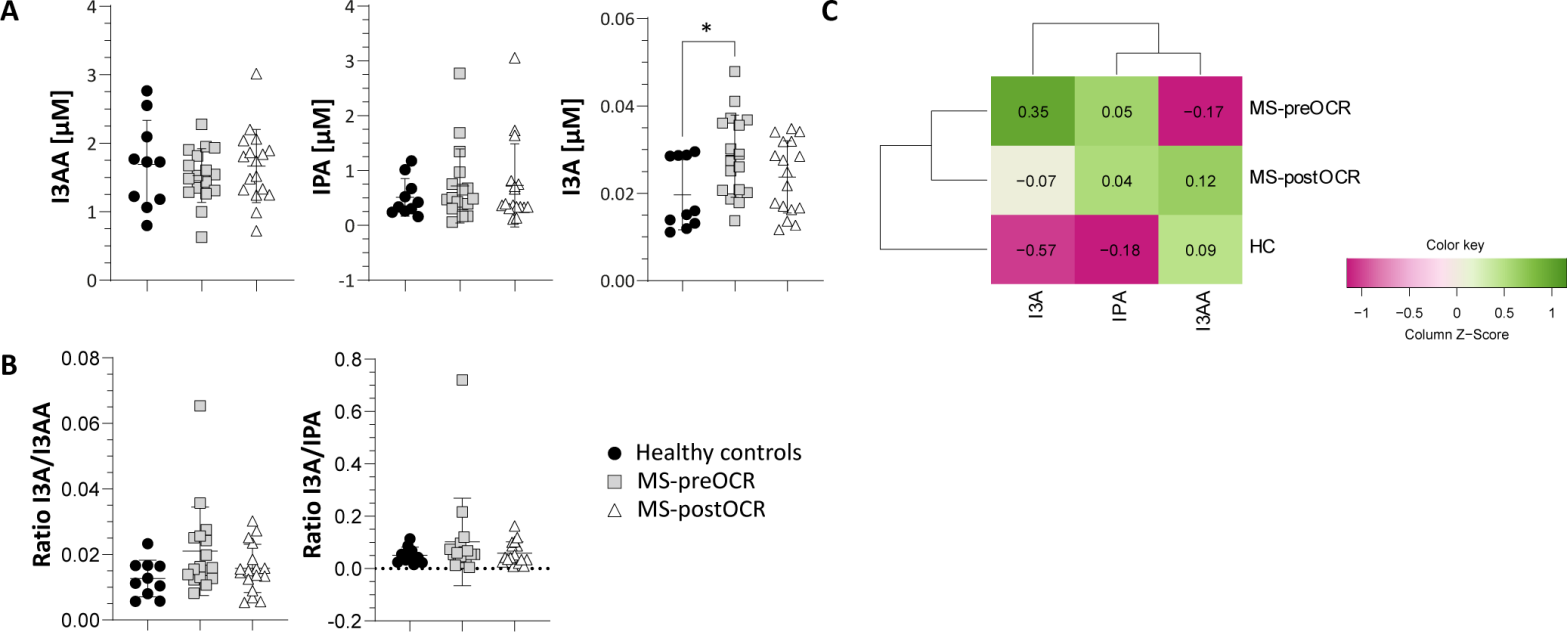
Evaluation of Indole pathway in HC and MS patients before and after OCR treatment. **(A)** Micromolar concentration [µM] of Ind metabolites (I3AA, IPA and I3A). **(B)** I3A/I3AA ratio to evaluate *Lactobacilli* capacity to produce I3A, I3A/IPA ratio to evaluate the contribution of *Lactobacilli* over C*lostridium* sp*orogenes*. **(C)** Heatmap including all Ind-related metabolites among HC and MS before and after OCR treatment. Heatmap’s rows indicate the condition, while the columns indicate the metabolites of Ind-related pathway. Dendrograms reporting clustering groups are displayed among rows and columns. The color-legend for z-score values is reported. MS patients (n= 17) before (MS-preOCR) and after (MS-postOCR) OCR treatment; Healthy controls (HC; n= 10). HC *vs* MS-preOCR or MS-postOCR: non-parametric Mann-Whitney unpaired test; MS-preOCR *vs* MS-postOCR: non-parametric paired test. *p<0.05.

Trp can be metabolized by microbiota to produce Ind pathway derivatives which are Tryptamine (Tryp), Indole-3-pyruvic Acid (I3PA), Indole-3-Acetic Acid (I3AA), Indole-3-Carboxaldehyde (I3A) and Indole-3-propionic Acid (IPA) (**Figure 1**). As shown in **Figure 4A**, we did not find any significant difference in circulating I3AA and IPA metabolites among the three groups. On the contrary, we detected a significant increase in I3A metabolite in MS-preOCR patients with respect to HC. Additionally, I3A/I3AA ratio (**Figure 4B**), which is indicative of *Lactobacilli* capacity to produce I3A, is slightly increased in MS-preOCR compared to HC and MS-postOCR although it does not reach the statistical significance (p=0.06 and p=0.134). However, I3A/IPA ratio (**Figure 4B**), which reflects the contribution of *Lactobacilli* over C*lostridium* sp*orogenes* in I3A production, is comparable in the three groups. Different from Kyn and 5-HT pathways, the overall analysis of Ind pathway clusters together MS patients and is not reverted after OCR treatment (**Figure 4C**).

### 3.4 Impairments in metabolites are observed in both female and male MS patients

**Figure 5:**
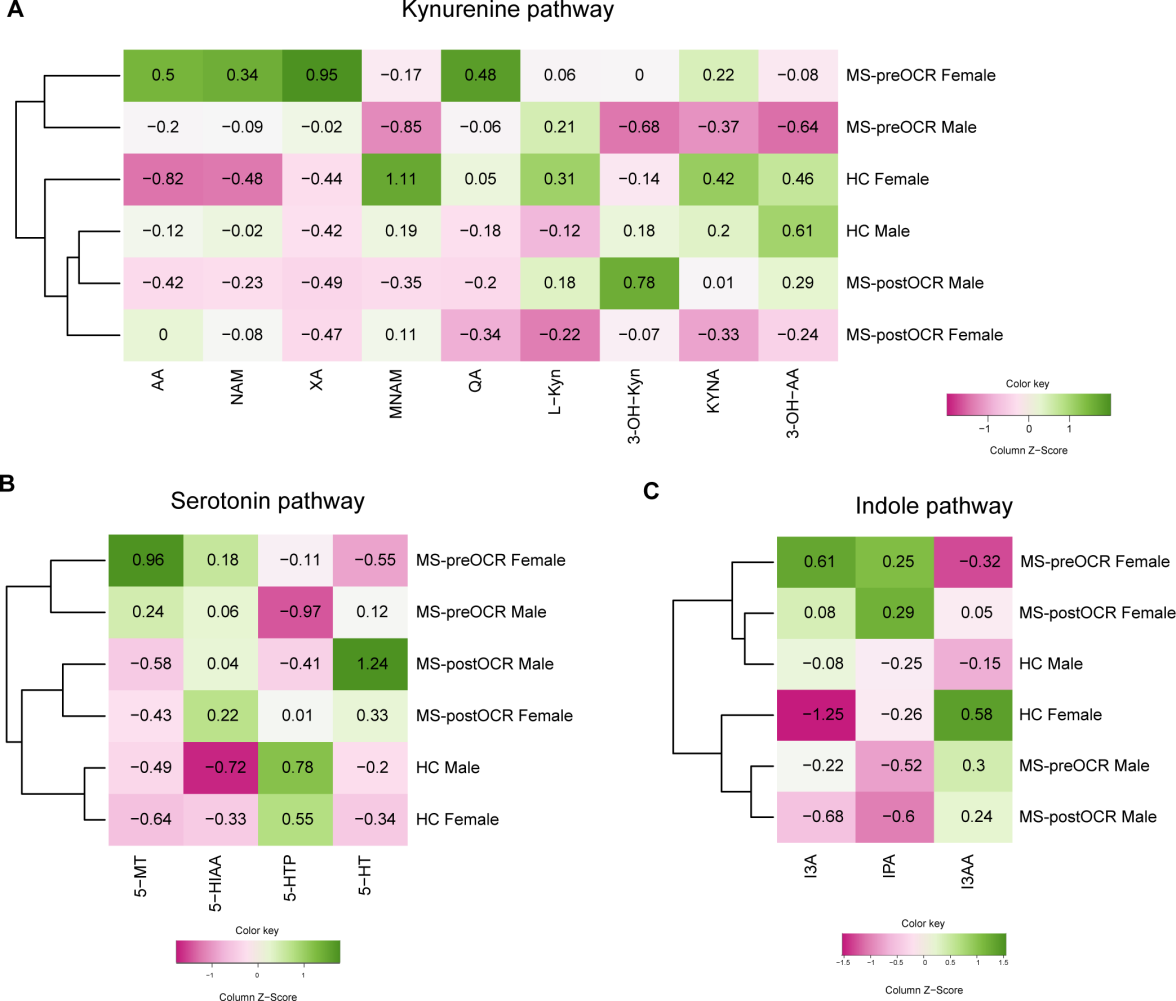
Sex differences in Trp metabolites. Heatmaps including all metabolites for each Trp-related pathway, stratified by sex among HC and MS before and after OCR treatment, are reported. divided by sex. Heatmap’s rows indicate the condition, while the columns indicate the metabolites of: **(A)** Kyn pathway, **(B)** 5-HT pathway, **(C)** Ind pathway. The color-legend for z-score values is reported.

Considering the differential disease incidence between males and females, we aimed to assess whether there are sex-specific variations in metabolite concentrations across the three distinct pathways. **Figure 5** presents three heatmaps, one for each pathway, illustrating the normalized data for each metabolite in HC, MS-preOCR and MS-postOCR, divided between male and female subjects.

The stratification of Kyn metabolites (**Figure 5A**) revealed a sex-independent clustering according to the three groups. We found that both HC and MS-postOCR cluster together, separately from MS-preOCR. A similar observation can be made regarding the 5-HT pathway (**Figure 5B**). These results reflect the clustering obtained by analyzing the data without stratification by sex (**Figures 2C**, **3C**), suggesting that untreated patients undergo a variation in the concentration of Kyn and 5-HT pathways’ metabolites, regardless of being males or females. Furthermore, it can be observed that the recovery of Kyn and 5-HT pathways to HC metabolite pattern is also independent of sex.

Conversely, the Ind pathway does not align with the clustering neither according to sex (**Figure 5C**) nor health status (**Figure 4C**). However, this does not exclude the absence of an influence of OCR treatment on gut microbiota composition. Rather, a more realistic evaluation of indoles composition should be implied by their quantification in feces as opposed to those in blood, which contains only the fraction that has been absorbed.

## 4 Discussion

MS is a chronic inflammatory autoimmune disease affecting the CNS characterized by a wide range of symptoms including fatigue, walking difficulties, muscle weakness and cognitive loss. MS can take different forms, with the most common being the RRMS characterized by exacerbation of symptoms followed by remission. In MS pathology, heightened inflammation can exert a substantial influence on numerous metabolic pathways that are implicated in the interactions between intestinal epithelial cells (IECs), gut-microbiota, and the immune system. Reducing inflammation is one of the benefits of B-cell depleting therapy with OCR in MS patients (24, 25). Based on this evidence, metabolic changes may also be reversed following treatment. In this view, given the involvement of Trp and its derivatives in the modulation of crucial processes including immune responses, neuronal and intestinal homeostasis (13), we decided to analyze the Trp metabolomic profile in RRMS patients before and after OCR treatment and compared the quantifications with the ones of HC. Some Trp metabolites, such as host derived Kyn and I3A, can act as ligands for the Aryl Hydrocarbon Receptor (AhR). Zelante et al. found that I3A levels inversely correlate with disease duration in MS patients. In turn, their results suggest how AhR regulates endogenous Trp catabolic pathways exerting a protective role when activated, highlighting how Trp-derived postbiotics can contribute to immune homeostasis (26, 27). Indeed, multiple studies indicate that dysregulated AhR signaling may influence the development and progression of MS by impacting immune cell functions, neuroinflammation, and the gut-brain axis (28).

**Figure 6:**
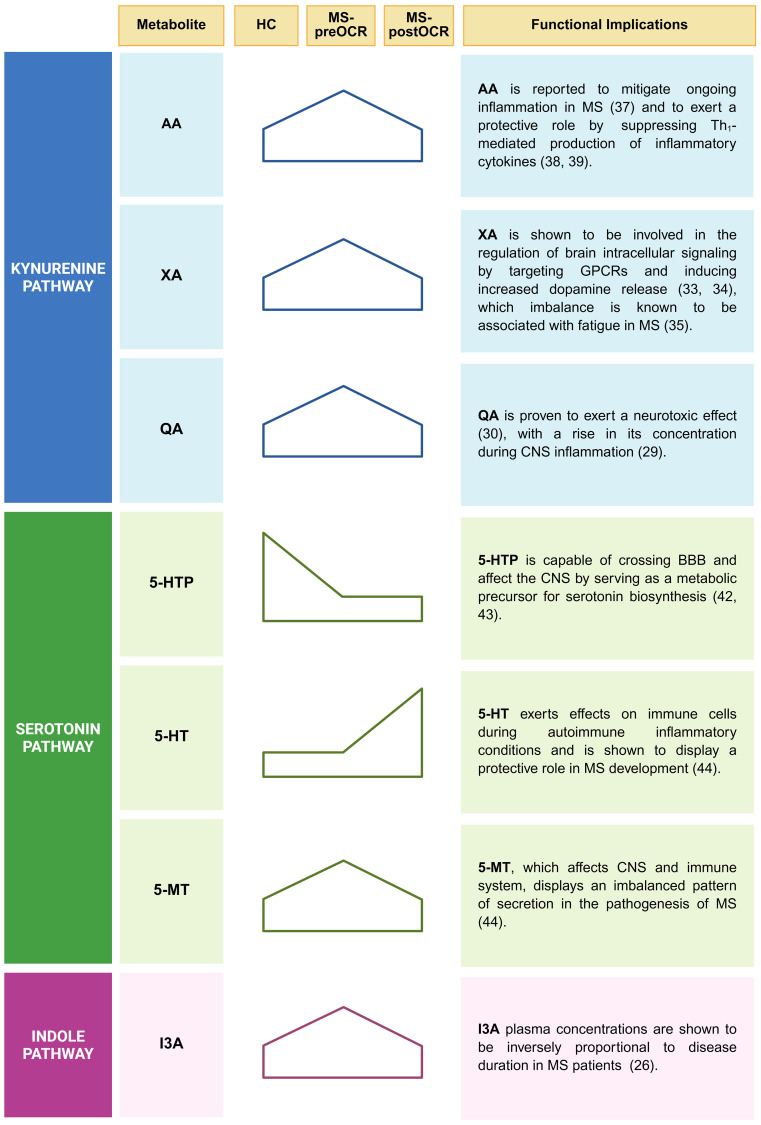
Principal alterations of Trp metabolomic profile among RRMS patients. A synthesis of the major alterations identified in the Kynurenine, Serotonin and Indole pathways are displayed. Both statistically significant or trend variations, for each corresponding metabolite, are shown. Functional implications in inflammatory context linked to MS are listed for each reported metabolite. Created in BioRender. Pivetta, M. (2025). https://BioRender.com/j8wliwt.

We found significant alterations of Trp metabolomic profile among RRMS patients (**Figure 6**). The Kyn pathway is known to be the main metabolic route for Trp degradation, followed by 5-HT and microbiota-dependent Ind pathways. Our analyses of Kyn metabolites showed an increase of QA, XA, and AA in MS untreated patients. Higher QA concentrations have been observed during CNS inflammation (29) and it has been proved that QA is neurotoxic (30). Similarly to QA, XA increases in untreated patients. Xanthurenic acid is an intermediate of the kynurenine pathway and is directly produced from 3-Hydroxykynurenine (3OH-Kyn) by the KAT2 (23). We described a significant increase of XA in untreated MS-patients: KAT2 is also expressed in red blood cells (RBCs) and, its activity is higher in RBCs of MS patients, that could be a possible explanation of the increase found (31, 32). The role of XA is not clear yet, but there are some evidences that this metabolite is capable to pass through the blood brain barrier (BBB) and can target G-protein coupled receptors (GPCRs) (33) and induce increased dopamine release (34). Of note, dopamine imbalance has been linked to cognitive impairments, fatigue and depression in MS and other neurological disorders (35). In parallel, Cinnabarinic acid (CA) is a “forgotten” kynurenine pathway metabolite of tryptophan, produced by the autoxidation of 3-OH-AA and, although we cannot quantify it in our conditions, we can hypothesize that its concentration is lower in MS-preOCR patients, since they preferentially produce XA and QA, as suggested by XA/3OH-AA and QA/3OH-AA ratios (**Figure 2B**). Interestingly, Fazio et al. showed that systemic administration of CA was protective against experimental autoimmune disease, suggesting that this metabolite can act against neuroinflammation (36). In regard of AA, also Pires et al. showed that RRMS patients have increased concentration of this metabolite. They hypothesized that in MS patients it could have a compensatory function in the mitigation of the ongoing inflammation (37). Furthermore, it has been shown that it has a protective role in MS suppressing production of pro-inflammatory cytokines by Th_1_ cells (38, 39). Overall, we found a general alteration of Kyn pathway metabolites that is partially restored following OCR, which suggests a positive effect of the therapeutic intervention, probably because of inflammation reduction.

In addition to Kyn pathway dysregulation, we identified alterations also in the 5-HT pathway. Serotonin is a key neurotransmitter in the brain-gut axis and for the 90% is produced by gut enterochromaffin cells that are influenced in their function by gut microbiota (17) and by inflammatory mediators, such as IL-33 (40, 41). While gut-derived 5-HT cannot pass the blood brain barrier, its precursor (5-HTP) and melatonin can, thus are capable to influence the CNS (42, 43). We showed that the end-product of the 5-HT pathway, 5-MT, is increased in MS untreated patients, while the 5-MT precursor 5-HT increases after treatment. This suggests an imbalance in this pathway, with much higher amounts of 5-HT being used to make 5-MT in MS patients compared with HC, an observation that is further supported by 5-MT/5-HT ratio. According to literature, altered 5-MT secretion characterizes MS patients (44). Therefore, it can be hypothesized that in MS, enterochromaffin cells augment the utilization of Trp, resulting in an increase of the end-product 5-MT. Reverchon et al. demonstrated an overexpression of the T lymphocyte serotonin 5-HT_7_ receptor in MS patients treated with natalizumab (NTZ), particularly following treatment (45). The study posited that the overexpression of the 5-HT_7_ receptor in MS treated patients was associated with an inflammatory context that was mitigated by NTZ. The same considerations regarding the decreased inflammatory context can be applied in the context of OCR (46). This suggests the potential for an increased 5-HT_7_ receptor on T lymphocytes in this patient population, which may consequently lead to an increased probability of 5-HT utilization, likely contributing to the observed increase in treated patients and concomitant decrease in 5-MT. The study also demonstrates a correlation between the increase in 5-HT_7_ and the increase in IL-10, which is an immune-regulatory cytokine (47). This finding suggests a beneficial effect of 5-HT utilization in the context of autoimmune diseases, given the immunoregulatory properties of IL-10.

Regarding the microbiota-dependent Ind pathway, it is important to highlight that we quantified indoles in the blood, thus only metabolites that are absorbed through the intestinal epithelium and not produced in the gut. We found that the concentration of the I3A metabolite is significantly higher in MS untreated patients, compared with HC and a trend decrease is present after OCR treatment, suggesting that there might be either deregulation in the gut barrier or changes in the microbiota. This last hypothesis is in line with the evidence that specific gut microbiome composition, different from healthy control, is associated to MS (5). Troci et al. demonstrated that B-cell depletion drugs for MS, including OCR, are associated with regression of dysbiosis: after B-cell depletion, an increase in alpha diversity within the gut microbiota occurs, accompanied by a protracted decline in pro-inflammatory bacteria (21). These findings could explain the dysregulation of I3A in untreated MS patients which returns to values comparable to HC following OCR treatment. Given the focus provided by our study on plasma metabolites quantification, it would be insightful to consider fecal indoles quantification and microbial profiling as future perspectives to further explore these results. This type of analysis can also allow to exclude possible interferences given by Trp intake.

Overall, these findings suggest a positive effect of OCR therapeutic intervention, which leads to a regression of metabolic dysregulations, probably as a consequence of inflammation reduction (24, 25).

Such decrease in inflammatory state could justify the shift towards a closer physiological metabolic profile: for example, inflammatory stimuli (e.g. LPS, IFNγ or TNFα) are reported to increase quinolinic acid concentration *in vitro* (48). Such results are reflected in our *in vivo* study for which we identified augmented QA levels in MS untreated patients, and after OCR treatment, they tend to diminish, in line with the reduction of inflammation. As well as OCR, also other therapies for MS aim to mitigate the inflammatory state: some studies have reported altered L-Kynurenine levels and Kyn/Trp ratios in MS patients during treatment with IFN-β (49) or glucocorticoids (50). Unlike the studies present in the literature regarding MS treatment and Trp metabolism, our research offers a broader and deeper overview about such relationships occurring in the OCR treatment context. Moreover, given the lack of data about the relationship between various MS treatments and their impact on Trp metabolism, our investigation provides also new possible landscapes to investigate about.

In addition, we showed that the imbalance and the subsequent restoration of Trp metabolism is maintained also across sexes. Although we analyzed a more limited number of healthy subjects compared to patients, the novelty of our study resides on the simultaneous characterization of three different Trp pathways, analyzed at the level of each metabolite involved, over RRMS patients before and after OCR infusion. Given the lack of notions about Trp metabolomic profile among RRMS OCR- treated patients, our data provides new perspectives among the relevancy of Trp in inflammatory and autoimmune diseases and represents a starting point for comprehending the effects provided by this drug on MS patient’s metabolism.

## Data Availability

All relevant data is contained within the article: The original contributions presented in the study are included in the article material, further inquiries can be directed to the corresponding author.
